# Metastasis of nasopharyngeal carcinoma to parotid lymph nodes: a retrospective study

**DOI:** 10.1186/1477-7819-13-1

**Published:** 2015-01-26

**Authors:** Shengye Wang, Jianlin Lou, Suzhan Zhang, Liang Guo, Kejing Wang, Minghua Ge

**Affiliations:** Department of Oncology, Second Affiliated Hospital, Zhejiang University School of Medicine, 88# Jiefang Road, Hangzhou, 310009 China; Department of Radiation Oncology, Zhejiang Cancer Hospital, 38# Guangji Road, Hangzhou, 310022 China; Department of Head and Neck Surgery, Zhejiang Cancer Hospital, 38# Guangji Road, Hangzhous, 310022 China

**Keywords:** Parotid node metastasis, Nasopharyngeal carcinoma, Neck dissection

## Abstract

**Background:**

Malignant parotid tumors are rare metastases originating from nasopharyngeal carcinoma (NPC). This study aimed to investigate the clinicopathological features and outcome of patients with metastasis of NPC to parotid lymph nodes after surgical therapy.

**Methods:**

We enrolled 14 NPC patients who had metastatic disease to parotid lymph nodes after IMRT. They received surgical treatment by total parotidectomy with neck dissection, superficial parotidectomy with neck dissection, partial parotidectomy with neck dissection, total parotidectomy, or superficial parotidectomy. Their age, gender, histopathology, clinical findings, and treatment outcome were analyzed.

**Results:**

After radiotherapy, parotid metastasis represented as uncontrolled disease in three cases and as recurrent disease in 11 cases. All the 14 patients received salvaged surgery successfully. Pathologic findings showed grade 3 in most patients. The follow-up ranged from 11 to 120 months and the overall three- and five-year survival was 49.5% and 37.1%, respectively.

**Conclusions:**

Metastasis to parotid lymph nodes should be examined in NPC patients after IMRT. Resection of the inferior parotid lymph nodes is recommended for patients with cervical metastasis, and superficial or total parotidectomy and adjuvant therapy are recommended for intraparotid lymph node metastasis.

## Background

A wide range of primary and metastatic neoplasms can present as masses in the parotid gland. Roughly a quarter of malignant parotid tumors are metastases originating from head and neck tumors such as scalp, face, eyelid, oral cavity, and oropharynx. Among malignant parotid tumors, squamous cell carcinoma (SCC) and malignant melanoma are the most common [1–3]. Nasopharyngeal carcinoma (NPC) has a high incidence in China, especially in the southeast.Malignant parotid tumors are rare metastases originating from NPC. Radiotherapy is the most standardized method for NPC and the conventional dose of radiotherapy is between 60 and 70 Gy.

The parotid space harbors three groups of lymph nodes determined by the embryological development of the parotid gland. The first group of lymph nodes are embedded within the parotid fascia, the second group in the parotid parenchyma, and the last group such as the preauricular lymph nodes remain extrafascial and extraglandular. Although metastases to parotid lymph nodes are uncommon, it should always be considered in the differential diagnosis of a parotid mass. In this study we aimed to review and investigate the clinicopathological features and outcome of patients with metastasis of NPC to the parotid lymph nodes.

## Methods

This study was conducted according to the Declaration of Helsinki on biomedical research involving human subjects. The study protocol was approved by Ethics Committee of Zhejiang Cancer Hospital. The approval number is zjzlyy[2014]-11-96. We retrospectively reviewed the records of patients with NPC in Zhejiang Cancer Hospital between January 2001 and December 2012. Among all 9,602 cases of NPC, 14 patients had metastasis of NPC to parotid lymph nodes after IMRT and received salvaged surgical therapy. In detail, all patients were asked to lie in the supine position and to wear a plastic head and neck mask. The primary tumor and whole neck were treated by IMRT. The total dose was 69 Gy in 30 fractions at 2.3 Gy/fraction to the primary gross target volume (PGTV), 66 Gy to the positive nodal gross target volume (GTV) node, 63 Gy to the planning target volume (PTV) node, 60 Gy to the high-risk neck regions, and 54 Gy to the low-risk neck regions. PGTV included GTV of nasopharynx lesion and retropharyngeal lymph nodes, plus a 3 to 5 mm margin. Treatment was delivered by a stationary, multileaf intensity-modulating collimator (Varian, Palo Alto, California, United States). All targets were treated simultaneously using the simultaneous integrated boost technique.

Patients were considered eligible for the radiation treatment if they had histologically confirmed NPC with metastasis to parotid lymph nodes, including the uncontrolled disease (indicating that residual tumors remain after IMRT) and delayed metastasis. Patients with primary parotid malignancies, lymphomas, history of a second primary tumor, direct tumor extension to the parotid gland or advanced untreatable disease were excluded from this study.

A total of 14 patients met the study criteria and their data such as the age, gender, histopathology, clinical findings, and treatment outcome were analyzed. The mean and median follow-up period after treatment of metastatic disease was 55.5 and 34.0 months, respectively (range: 11 to 120 months). Survival analysis was performed using the Kaplan-Meier method.

## Results

The characteristics of the 14 patients were shown in Table 1. The patients included 12 male and two female. Age ranged from 18 to 74 years (mean: 50.4 years). The pathological diagnosis of the primary tumors was grade 3 (poorly differentiated carcinoma) in most of the patients. A total of three patients were identified as uncontrolled disease of parotid metastasis because lesions recurred within three months after the initial treatment of radiotherapy. Among the delayed metastasis of parotid group, the mean time interval between the treatment of the primary tumor and the diagnosis of metastatic disease to the parotid gland was 55.9 months (range: 15 to 108 months). All patients presented with a clinically palpable parotid metastatic mass on physical examination. A total of six patients (42.9%) had additional enlarged cervical lymph nodes. The most common location of metastases within the parotid gland was the inferior or superficial parotid nodes, and four patients had positive nodes in both the superficial and deep parotid nodes.

**Table 1:** **The clinical data on metastasis of nasopharyngeal carcinoma cancer to parotid lymph nodes**

Gender	Age	Pathology and grading	Parotid metastasis	Treatment of primary tumor	Treatment of metastasis	Surgery mode	Parotid lymph node metastasis	Size	Facial nerve paralysis	Outcome
Female	45	SCC, G3	Uncontrolled	CRT	S	SP	superficial	<3 cm	-	M
										AWD
										15 months
Male	46	SCC, G3	Delayed 15 months	RT	S	SP	superficial	<3 cm	-	ANED
										97 months
Male	44	SCC, G3	Delayed 47 months	CRT	S	TP	superficial deep	>3 cm	-	M
										DOD
										53 months
Male	74	SCC, G3	Delayed 60 months	CRT	S	PP + ND	inferior superficial	<3 cm	-	DOD
										24 months
Female	46	SCC, G3	Delayed 108 months	CRT	S	SP + ND	superficial	>3 cm	+	ANED
										17 months
Male	51	SCC, G3	Delayed 105 months	CRT	S	TP + ND	superficial deep	<3 cm	+	DOD
										28 months
Male	57	SCC, G3	Delayed 36 months	RT	S + RT	TP	superficial deep	>3 cm	+	LR
										AWD
										38 months
Male	32	SCC, G3	Uncontrolled	CRT	S + RT	PP + ND	inferior	>3 cm	-	M
										25 months
										AWD
Male	18	SCC, G3	Uncontrolled	CRT	S + RT	SP + ND	superficial	<3 cm	-	M
										DOD
										65 months
Male	56	SCC, G3	Delayed 60 months	CRT	S + RT	SP + ND	inferior superficial	>3 cm	-	LR
										DOD
										11 months
Male	57	SCC, G3	Delayed 23 months	CRT	S + RT	TP + ND	inferior superficial	>3 cm	-	DOD
										13 months
Male	52	SCC, G3	Delayed 65 months	RT	S + CRT	SP + ND	superficial	<3 cm	-	DOD
										34 months
Male	67	SCC, G3	Delayed 72 months	RT	S + CRT	TP + ND	superficial	>3 cm	+	LR
										ANED
										120 months
male	61	SCC, G3	Delayed 24 months	CRT	S + CRT	TP + ND	superficial deep	>3 cm	+	M
										DOD
										17 months

ANED: Alive with no evidence of disease; AWD: Alive with disease; CRT: Chemotherapy and radiotherapy; DOD: Dead of disease; G: Grading; LR: Local recurrence; M: Distant metastasis; ND: Neck dissection; PP: Partial parotidectomy; RT: Radiotherapy; S: Surgery; SCC: Squamous cell carcinoma; SP: Superficial parotidectomy; TP: Total parotidectomy.

**Figure 1:**
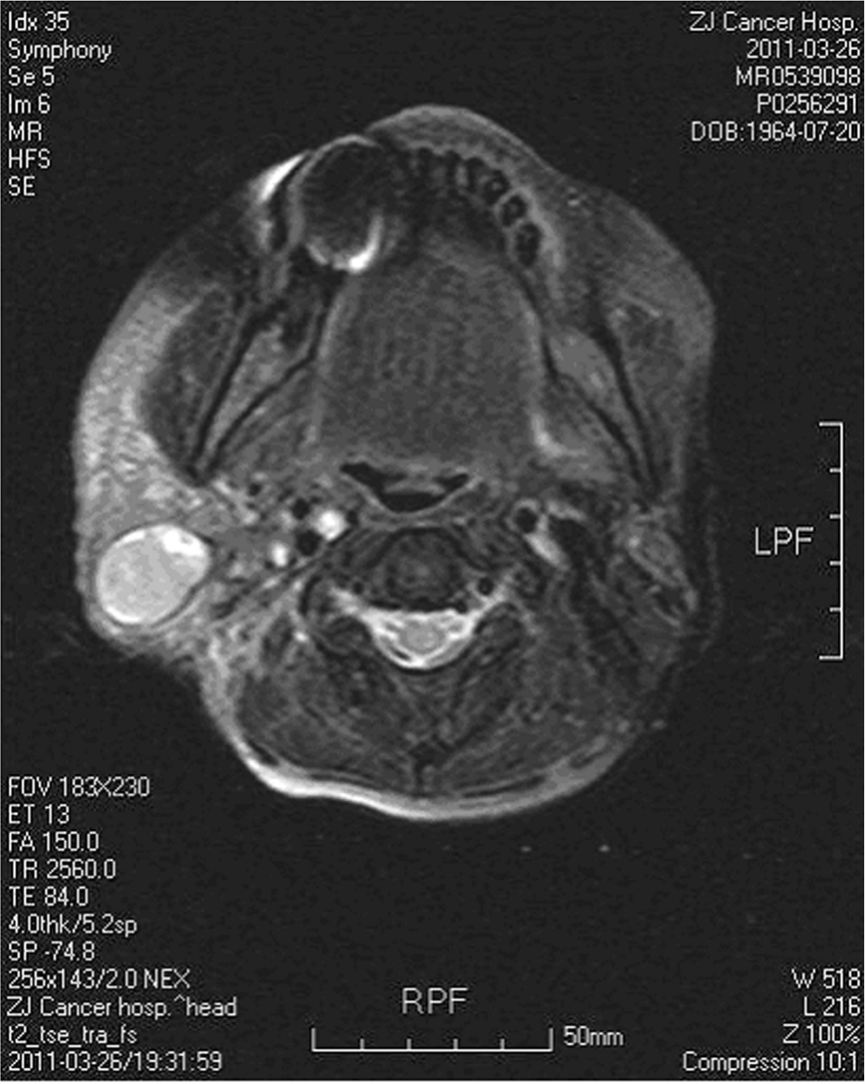
**MRI image of uncontrolled parotid metastasis of nasopharyngeal carcinoma after radiotherapy.** Multiple swollen lymph nodes were observed in bilateral carotid sheath and pharyngeal lymph node area.

All 14 patients received IMRT and/or chemotherapy as their initial treatment. Magnetic resonance imaging (MRI) confirmed uncontrolled parotid metastasis parotid metastasis in three patients and the typical MRI image for one patient is shown in Figure 1.

**Figure 2:**
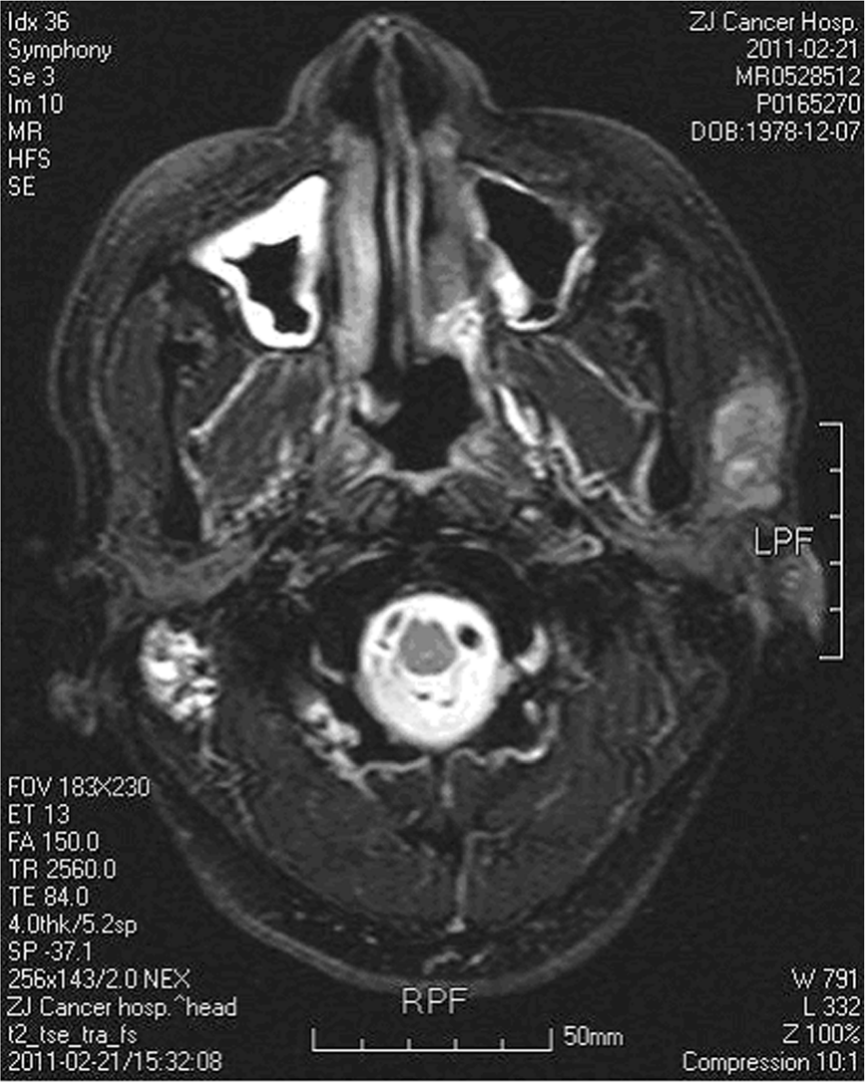
**MRI image of delayed parotid metastasis of nasopharyngeal carcinoma after radiotherapy.** A nodule about 2.4 × 1.4 cm was observed in the left parotid gland.

For the other 11 patients, MRI imaging confirmed delayed parotid metastasis and the typical MRI image for one patient is shown in Figure 2. Among the 11 patients presenting with delayed parotid metastasis, three cases underwent surgery and adjuvant radiotherapy, three cases underwent surgery, adjuvant radiotherapy, and/or chemotherapy, and five cases underwent surgery alone. The surgery procedure for the treatment of parotid metastasis was as follows: total parotidectomy with neck dissection, superficial parotidectomy with neck dissection, partial parotidectomy with neck dissection, total parotidectomy, and superficial parotidectomy.

**Figure 3:**
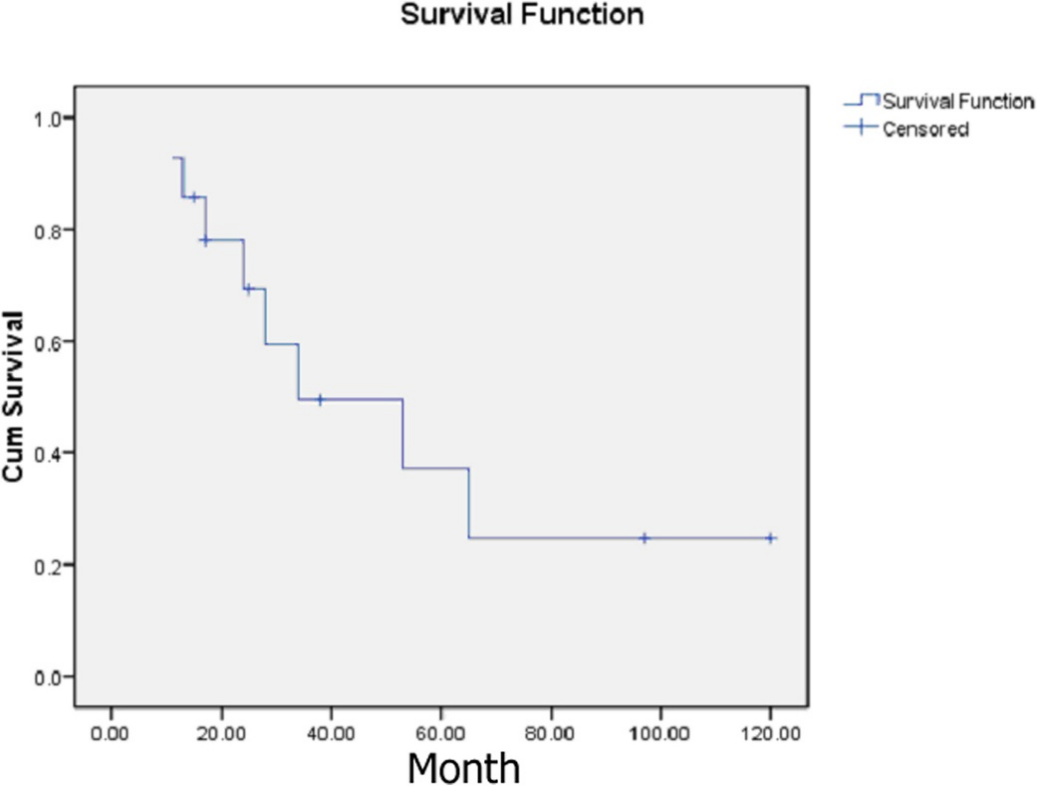
**Kaplan-Meier curve estimating the post-operative overall survival of 14 patients presenting with metastasis of nasopharyngeal carcinoma to parotid lymph nodes.**

Postoperative complications included facial paralysis or partial facial paralysis, parotid fistula, and flap necrosis. At the last follow-up assessment after treatment of parotid lymph nodes, tumor recurrence occurred in three patients and five patients showed distant metastasis. A total of eight patients died of disease, three patients were alive with no evidence of disease, and three patients were alive with disease. The overall three- and five-year cumulative survival was 49.5% and 37.1%, respectively. The medium survival was 34 months (Figure 3).

## Discussion

The incidence of NPC is high in China, especially in the southeast, including the Zhejiang province. Radiotherapy is the main treatment method for NPC and the usual dose of radiotherapy is between 60 and 70 Gy. With the development of IMRT, the protocols for NPC radiotherapy have been improved. To prevent the occurrence of xerostomia, the dose of IMRT is regulated. Normally, the dose for the radiation of parotid gland is limited to V26 to 30 < 50%. However, for any radiotherapy technique that involves facial and cervical joint field and auriculotemporal field in the facial and cervical regions, the dose for radiation of the parotid gland is higher than that in IMRT. This may explain why the incidence of NPC metastasis to parotid gland has recently increased with the application of IMRT.

The parotid lymphatics are known to receive drainage from a wide region of the head and neck [4, 5]. Parotid nodes are divided into superficial and deep groups based on the relation to facial nerve. Lymph nodes are present in superficial and deep lobes of the gland. The superficial and deep lobe has 3.9 to 7.6 and 1.05 to 2.3 lymph nodes, respectively [6, 7]. These intraparotid lymphatics render the parotid uniquely susceptible to tumor metastasis [8]. However, pathological findings have shown that all the parotid lymph nodes lie lateral to the retromandibular vein and not the facial nerve, thus any dissection that follows the nerve may leave residual disease because of anatomical variation in the position of the facial vein relative to the nerve [9].

Pathologic findings showed grade 3 tumors in most of the patients. We observed the correlation of increased risk of parotid node involvement with an increased number of cancer-positive cervical lymph nodes, consistent with a previous study [10].

Due to parotid node metastasis, it is important to have a careful clinical and radiographic evaluation of the parotid region in a patient with NPC cancer and cervical metastasis. During neck dissection, careful intraoperative inspection and palpation of the parotid should be performed. We recommend the removal of parotid tail nodes if metastatic involvement is suspected. There is still controversy on the selection of surgery to treat metastasis to parotid lymph nodes; while some surgeons prefer superficial parotidectomy, others prefer total parotidectomy [11]. Facial nerve preservation should be attempted unless the nerve is grossly involved with tumor.

In the present study, amongst the six patients who underwent a total parotidectomy, four were diagnosed with metastasis within the deep lobe. Therefore, we think that total parotidectomy is necessary due to the poor prognosis of untreated deep-lobe metastasis, although it could increase the risk of temporary facial nerve dysfunction.

Metastatic disease to the parotid gland is mostly caused by SCC of the head and neck, and long-term survival remains poor despite combined treatment modalities [12]. Several studies reported positive surgical margins, extranodal spread, advanced tumor stage, and the absence of adjuvant irradiation as factors of poor locoregional control [13, 14]. Combination therapy with surgery and radiotherapy has been shown to be effective to improve the survival of patients with advanced head and neck cancer [14, 15]. In terms of timing, surgery is considered the first and best option for patients deemed suitable for that, after a careful pre-operative risk-assessment. In cases of extranodal spread and advanced tumor stage, radiotherapy and/or chemotherapy are needed.

## Conclusions

The application of IMRT should be taken into account for metastatic disease to the parotid gland. Metastasis to the parotid lymph nodes should be examined in patients with NPC before initial treatment and after IMRT. For patients with substantial cervical metastasis, we recommend the resection of the tail of the parotid gland and the inferior parotid lymph nodes during the neck dissection. If intraparotid lymph node metastases are detected, we recommend total parotidectomy and adjuvant therapy.

## Abbreviations

GTV: gross target volume
IMRT: intensity-modulated radiation therapy
MRI: Magnetic resonance imaging
NPC: nasopharyngeal carcinoma
PGTV: primary gross target volume
PTV: planning target volume
SCC: squamous cell carcinoma.
